# Plasticity of GABA_A_ receptor diffusion dynamics at the axon initial segment

**DOI:** 10.3389/fncel.2014.00151

**Published:** 2014-06-10

**Authors:** James Muir, Josef T. Kittler

**Affiliations:** Department of Neuroscience, Physiology and Pharmacology, University College LondonLondon, UK

**Keywords:** gaba receptors, homeostatic plasticity, axon initial segment, calcium, quantum dots, diffusion

## Abstract

The axon initial segment (AIS), a site of action potential initiation, undergoes activity-dependent homeostatic repositioning to fine-tune neuronal activity. However, little is known about the behavior of GABA_A_ receptors (GABA_A_Rs) at synapses made onto the axon and especially the AIS. Here, we study the clustering and lateral diffusion of GABA_A_Rs in the AIS under baseline conditions, and find that GABA_A_R lateral mobility is lower in the AIS than dendrites. We find differences in axonal clustering and lateral mobility between GABA_A_Rs containing the α1 or α2 subunits, which are known to localize differentially to the AIS. Interestingly, we find that chronic activity driving AIS repositioning does not alter GABAergic synapse location along the axon, but decreases GABA_A_R cluster size at the AIS. Moreover, in response to chronic depolarization, GABA_A_R diffusion is strikingly increased in the AIS, and not in dendrites, and this is coupled with a decrease in synaptic residency time of GABA_A_Rs at the AIS. We also demonstrate that activation of L-type voltage-gated calcium channels is important for regulating GABA_A_R lateral mobility at the AIS during chronic depolarization. Modulation of GABA_A_R diffusion dynamics at the AIS in response to prolonged activity may be a novel mechanism for regulating GABAergic control of information processing.

## Introduction

The axon initial segment (AIS), a neuronal subdomain enriched with ion channels, scaffolding components and cytoskeletal elements, serves as a key site for action potential initiation, and separates neuronal input and output domains (Rasband, 2010). Several proteins, including Na^+^ channels, the scaffolds ankyrin-G (ankG) and β IV-spectrin, and the cellular adhesion molecule neurofascin 186, form a protein-dense segment of approximately 20 μm in length, located near to the cell soma (Rasband, 2010). The AIS can also translocate away from the cell soma in response to altered neuronal activity patterns (elevated extracellular K^+^, Grubb and Burrone, 2010), with all AIS proteins tested (including ankG, NaV channels and NF 186) found to undergo a distal shift of approximately 10 μm along the axon. This structural plasticity, which depends on activation of voltage-gated calcium channels, results homeostatically in increased thresholds for action potential firing (Grubb and Burrone, 2010; O'Leary et al., 2010).

The AIS also receives GABAergic input from axo-axonic interneurons, which contact AIS-localized postsynapses containing clusters of GABA_A_Rs, while other neurotransmitter receptors are primarily absent from the AIS (Kole and Stuart, 2012). GABA_A_Rs are the major mediators of fast synaptic inhibition in the brain, though evidence suggests that axo-axonic inputs can also be depolarizing or excitatory (Szabadics et al., 2006; Khirug et al., 2008; Kole and Stuart, 2012), thus possibly providing a dual function. It is clear that synapses made onto the AIS can control cell excitability, firing frequency and input–output relationship (Klausberger and Somogyi, 2008; Kole and Stuart, 2012). GABA_A_Rs containing α1, α2, or α3 subunits are found enriched at synapses while α4, α5, and α6 are found primarily extrasynaptically (Luscher et al., 2011). Of the synaptic α subunits, α2 subunits (and α3 in some cell types) are enriched at the AIS, while few GABA_A_Rs at the AIS contain the α1 subunit (Nusser et al., 1996; Brünig et al., 2001; Panzanelli et al., 2011). While GABA_A_R membrane dynamics have been well studied in dendrites, including their lateral diffusion into and out of synapses (Thomas et al., 2005; Bannai et al., 2009; Muir et al., 2010), virtually nothing is known about the clustering and lateral mobility of GABA_A_Rs at the AIS. Moreover, whether GABAergic AIS synapses shift away from the soma in response to chronic depolarization, or whether the diffusion dynamics of GABA_A_Rs at the AIS can be modified is unknown.

Here, we investigate the subunit-specific differences between α1- and α2- containing GABA_A_Rs in terms of their clustering and lateral mobility at the AIS. We find that α2 clusters are more numerous in the axon than α1 clusters, and that GABA_A_R lateral mobility at the AIS is lower for α2- vs. α1-containing GABA_A_Rs. While the AIS moves away from the cell body in response to chronic depolarization, GABAergic pre- and postsynaptic elements remain fixed in position along the axon. In contrast, GABA_A_R lateral mobility in the AIS and proximal axon is specifically increased in response to chronic depolarization, coupled with decreased residency time at AIS synapses and reduced GABA_A_R cluster size in the AIS. Increased AIS-GABA_A_R lateral mobility is caused by activation of L-type VGCCs, which also drives AIS translocation (Grubb and Burrone, 2010). Our results provide a novel mechanism for modulation of GABAergic synapses under conditions of prolonged activity, which could have important implications for control of neuronal activity and information processing.

## Materials and methods

### Cell culture and drug treatments

We used standard culture of primary dissociated hippocampal neurons from E18 embryonic rats as described previously (Banker and Goslin, 1991). For chronic depolarization, the extracellular potassium concentration was elevated from 5 to 15 mM by adding KCl from a 1M stock solution. Nifedipine was from Tocris. KCl (15 mM) and nifedipine (5 μM) treatments were made at 12 DIV for 48 h, and all experiments were performed at 14 DIV. Transfection of ankG-GFP (a kind gift from V. Bennett, HHMI) was made by calcium phosphate precipitation at 10 DIV as previously described (Twelvetrees et al., 2010). Transfection of mGFP was made by lipofectamine 2000 (Invitrogen) at 11 DIV, with 72 h expression before staining.

### Live-cell imaging

Imaging media used for quantum dot tracking experiments (Muir et al., 2010) contained 125 mM NaCl, 5 mM KCl, 1 mM MgCl_2_, 2 mM CaCl_2_, 10 mM D-glucose, 10 mM HEPES and was adjusted to pH 7.4 with NaOH before use. Cells were imaged under perfusion (4 ml/min) and heating (35–37°C). Fluorescence was captured using an Olympus microscope (BX51WI) with a 60x Olympus objective coupled to an EM-CCD camera (Ixon, Andor). Excitation was provided by a mercury spiked xenon arc lamp (Cairn). Appropriate filters were chosen for QDs, alexa dyes, and FM 4–64.

Live labeling of the AIS (Schafer et al., 2009) was performed by mixing 1 μl of anti- pan-neurofascin (pan-NF, neuromab) with 0.35 μl of anti-mouse alexa 488 (Invitrogen). This mixture was incubated on ice for 15 min to allow coupling. Then, 100 μl block solution (imaging media containing 10% horse serum) was added and the solution kept at room temperature (RT) for 2–3 min. For parallel QD labeling of GABA_A_Rs, rabbit anti- α1 or α2 (1:100, Synaptic Systems, both recognizing extracellular epitopes) was added to the pan-NF/alexa solution. Coverslips were incubated for 8 min at RT by inverting onto this solution spotted on film. Quantum dots (anti-rabbit 605 nm QD, 0.5 nM, Invitrogen) were attached with a subsequent 2 min labeling step in block solution, as above. Coverslips were washed 6–8 times in imaging media after each step. QD movies were of 200 frames, acquired at 8.5 Hz (movie length = 23.5 s). To minimize the amount of GABA_A_R internalization within the recording period, movies were recorded within 15 min of QD labeling. Labeling of active presynaptic terminals with FM 4–64 (Invitrogen) was performed by 1 min incubations in 1 ml imaging media, first with 1 μm FM 4–64 + 60 mM KCl, followed by 0.2 μm FM 4–64. Coverslips were then washed extensively before imaging.

### Fixed-cell imaging

Co-staining for ankG and GABAergic synapse components (α1 and α2-GABA_A_Rs, gephyrin, VGAT, all primary antibodies from Synaptic Systems, except ankG, neuroMab and γ2, a kind gift from J. M. Fritschy) was performed using standard immunofluorescence techniques. All primary antibodies were used at 1:100 with secondary staining at 1:500 with alexa 488/594 or cy5. Surface staining of GABA_A_Rs (α1 or α2, both extracellular epitope) was made with an initial step in block solution lacking detergent. For analysis of AIS and cluster position, approximately 30 neurons were analysed per condition from images of the whole cell including 100 μm of axon (zoom = 0.7). For analysis of GABA_A_R cluster size, images at 4 × zoom (25 μm length) were taken of the AIS and two regions of proximal dendrite chosen at random for each cell, and approximately 15 neurons per condition were imaged. All settings were kept constant across experiments. Confocal imaging was performed with Zeiss Pascal and Zeiss 700 microscopes equipped with 63× plan Apochromat oil objectives (NA 1.4).

### Image analysis

AIS position was measured from ankG staining using an automated detection routine similar to that used in (Grubb and Burrone, 2010). Briefly, the axon was traced in ImageJ (NIH) and straightened using the “Straighten” plugin. A running average of ankG intensity along the axon was made (window, *W* = 20 pixels approximately 5 μm). This image was then scaled such that its pixel intensities range from 0 to 1. Starting from the soma edge (*x* = 0), AIS start and end positions are defined as where the scaled ankG intensity first exceeds 0.33 and then drops below 0.33, respectively. To account for the size of the smoothing window, *W*/2 (approximately 2.5 μm) was added to output values of AIS start and end position. Good agreement was found between AIS start and length measurements as determined by this routine compared to analysis by manual inspection.

For analysis of the position of GABAergic synapse components along axons, the straightened image of the axon (as above) was used. Manual logging of each cluster position along the axon (up to 100 μm from the cell soma) was performed in ImageJ. Clusters were defined to be on the axon if their position overlapped with ankG staining and were classified as being before or within the AIS by manual inspection of cluster and AIS position (from ankG staining). GABA_A_R cluster size was analysed using the “Analyse Particles” function in ImageJ. AIS and dendrite images were first intensity-thresholded (constant across experiments). AIS clusters were classified as those overlapping with AIS/ankG staining.

Analysis of QD-GABA_A_R trajectories was performed as previously described (Muir et al., 2010) using custom detection and tracking software written in *Mathematica* (Wolfram Research). Instantaneous diffusion coefficients were calculated from the squared displacement across sequential trajectory segments of five frames, using the 2D diffusion relation, <x^2^> = 4Dt. Diffusion coefficients were then pooled within like groups/conditions. To analyse QD-GABA_A_R dynamics in different neuronal regions (i.e., AIS, proximal axon or sample dendrites), neuronal regions were first identified according to neurofascin staining and morphology and then isolated in ImageJ using the selection brush tool. For analysis of GABA_A_R diffusion in synapses, QD-GABA_A_Rs were defined as synaptic if within 0.75 μm of the center of FM 4–64 puncta. GABA_A_R residency time for each cell was given by the mean duration of QD-GABA_A_R trajectory segments during which the particle was diffusing within a synapse (defined as above).

To determine AIS and dendrite diameter, confocal images of 25 μm regions (zoom factor 4) were taken. Processes were straightened using the ImageJ Straighten plugin and then thresholded at the same value. To obtain the diameter of the process, the thresholded area was divided by the image length (25 μm).

### Statistical analysis

All experiments were performed on neurons from at least 3 individual preparations. Unless otherwise stated, *p*-values given are from two-tailed Student's *t*-tests (equal variance) and values are given as mean ± s.e.m. Error bars represent s.e.m. For multiple comparisons (i.e., Figures 5A–C), One-Way ANOVA followed by Bonferroni correction was used. GABAergic synapse position along axons and GABA_A_R diffusion coefficients were not normally-distributed. Differences between conditions in these quantities were tested using the non-parametric Mann-Whitney *U*-test (implemented in *R*).

## Results

### GABA_A_R clustering and lateral mobility at the axon initial segment

**Figure 1:**
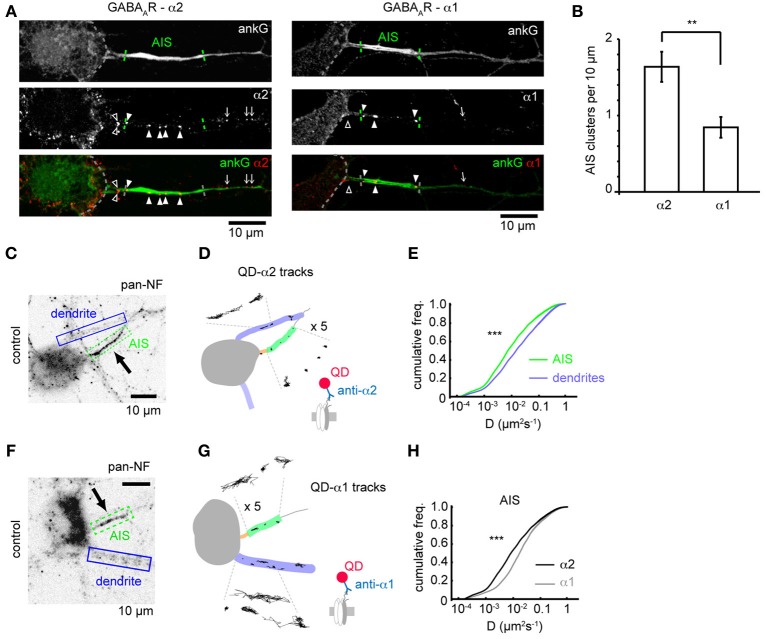
**GABA_A_R clustering and lateral mobility at the axon initial segment. (A)** Example neurons stained for ankG and the GABA_A_R α2 (left) and α1 subunits (right). Outline of soma, AIS position and gephyrin cluster positions along the axon are indicated (open arrowhead, cluster before AIS; closed arrowhead, cluster within AIS; arrow, cluster beyond AIS). **(B)** Clusters (normalized per 10μ M of AIS length) of the α2 subunit are more numerous in the AIS than the clusters of the α1 subunit, (*p* = 0.01, *n* = 5 experiments). **(C)** Neurofascin (pan-NF) staining delineates the AIS (green). Dendrites (blue) are also identified. **(D)** Subdomains in **(C)** overlaid with QD-α2 tracks (also shown zoomed in × 5 for clarity). **(E)** Cumulative frequency plots of instantaneous α2-GABA_A_R diffusion coefficient in AIS (green, *n* = 40,681) and dendrites (blue, *n* = 316,774); 97 cells, 13 experiments, (*p* < 2 × 10^−16^). **(F,G)** As in **(C,D)** for QD-α1 labeling. **(H)** α1- and α2-GABA_A_R diffusion coefficients in the AIS (gray, α1, *n* = 10,999, 45 cells; black, α2, *n* = 40,681, 97 cells, 13 experiments). α2-GABA_A_Rs are less mobile in the AIS than α1-GABA_A_Rs (median D; α 2 = 0.008 μm^2^s^−1^, α 1 = 0.014 μm^2^s^−1^, *p* < 2 × 10^−16^).

Subunit composition of GABA_A_Rs is key to determining their subcellular localization. Previous studies have shown that GABA_A_Rs containing the α2 subunit are preferentially targeted to the AIS compared to those containing the α1 subunit (Nusser et al., 1996) but whether this is due to subunit-specific differences in receptor diffusion dynamics remains unknown. We investigated the surface clustering and diffusion dynamics of GABA_A_Rs at the AIS containing either the α1 or α2 subunits using both immunofluorescence and single particle tracking. We surface stained with antibodies to either α1 or α2 subunits and co-stained for ankG to mark the AIS. Both α1 and α2 subunits were found clustered in dendrites as previously described (Nusser et al., 1996; Brünig et al., 2001). GABA_A_Rs containing the α2 subunit were also routinely found in clusters along axons (Figure 1A), while α1 was seen to be more diffuse, but exhibited a clustered distribution in approximately 20% of neurons, (which are likely interneurons, Brünig et al., 2001). In these cells, limited α1 clustering along axons could be seen (Figure 1A). In agreement with the literature (Nusser et al., 1996), we found that α2 clusters were far more numerous in the AIS than α1 clusters (α2: 3.6 ± 0.4, α1: 1.6 ± 0.3, *p* = 0.002, Figure 1B; quantification was from neurons exhibiting clustered GABA_A_R distribution only), confirming that the α2 subunit is enriched at the AIS compared to α1.

We then used single-particle tracking with quantum dots to investigate the lateral mobility of GABA_A_Rs, combined with neurofascin live-labeling (Schafer et al., 2009), to mark the AIS, which reliably labels the AIS as seen by comparison with ankG-GFP expression (Supplementary Figure 1). As expected, QD-α2-GABA_A_R labeling was seen in the AIS, axon and somatodendritic region (Figures 1C,D). Interestingly, α2-GABA_A_R lateral mobility was much lower in the AIS than in dendrites (AIS: median *D* = 0.008 μm^2^s^−1^, dendrites: median *D* = 0.016, *p* < 2 × 10^−16^, Figure 1E), as has previously been observed for lipid diffusion (Nakada et al., 2003). Recent studies suggest that the diameter of a tubular membrane can affect diffusion measurements (Renner et al., 2011). To assess whether differences in AIS and dendrite diameter could explain the difference in GABA_A_R diffusion between these two compartments, we used transfection of membrane GFP (mGFP) and ankG immunostaining to quantify AIS and dendrite diameter (Supplementary Figure 2). We found that typical AIS and dendrite diameters were both approximately 1 μm, and were not significantly different (*p* = 0.5), confirming that different tubular diameter could not account for observed differences in AIS and dendritic GABA_A_R diffusion.

We also analysed the diffusion dynamics of GABA_A_Rs containing the α1 subunit (Figures 1F,G). Interestingly, while α1-GABA_A_Rs were less mobile at the AIS than dendrites (AIS: median *D* = 0.014 μm^2^s^−1^, dendrites: median *D* = 0.022 μm^2^s^−1^), they were much more mobile than α2-GABA_A_Rs, particularly at the AIS (median D; α 1 = 0.014 μm^2^s^−1^, α 2 = 0.008 μm^2^s^−1^, *p* < 2 × 10^−16^, Mann-Whitney *U*-test, Figure 1H), but also in dendrites (median D; α 1 = 0.022 μm^2^s^−1^, α 2 = 0.016 μm^2^s^−1^). Using the ratio of median *D*-values (dendrite/AIS) as a measure of lateral mobility restriction in the AIS suggests that α2-GABA_A_Rs are more stable in the AIS membrane than their α1-containing counterparts (median D_dend_/D_AIS_: α 1 = 1.6, α 2 = 2.0), which likely underpins the enriched expression of α2 subunit-containing GABA_A_Rs observed in this region.

### The AIS shifts distally on chronic depolarization, but gabaergic synapse positions are not affected

**Figure 2:**
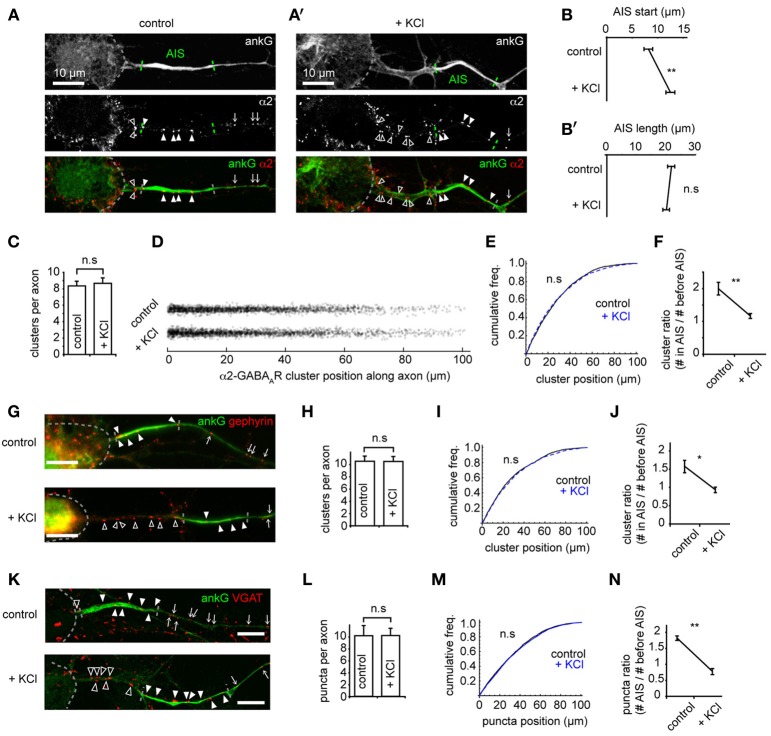
**The AIS shifts distally on chronic depolarization, but GABAergic synapse positions are not affected. (A)** As Figure 1A. Neuron stained for ankG and GABA_A_R-α2 subunit. Soma, AIS and GABA_A_R cluster positions along the axon are indicated (open arrowhead: cluster before AIS; closed arrowhead: cluster within AIS; arrow: cluster beyond AIS). AIS endpoints are determined from ankG intensity. **(A')** as (**A)**, for a KCl-treated neuron. **(B)** AIS start position is greater after KCl treatment (*p* = 0.007, *n* = 10 experiments); **(B')** AIS length is not affected (*p* > 0.05). **(C)** Number of GABA_A_R clusters per axon is unchanged (*p* > 0.05). **(D)** Positions of all GABA_A_R clusters from control and KCl pools. **(E)** GABA_A_R cluster position along axons (control, black, *n* = 1944 clusters; KCl, blue, dashed, *n* = 2130 clusters, *p* > 0.05, Mann-Whitney *U*-test). **(F)** Cluster ratio (in AIS / before AIS) is lower in KCl-treated neurons, *p* = 0.004, *n* = 5 experiments. **(G)** Example neurons stained for ankG and gephyrin (labeled as in **A**). Top: control, bottom: KCl. Scale bar = 10 μm. **(H)** Total number of gephyrin clusters per axon is not significantly different (*p* > 0.05, *n* = 5). **(I)** Cumulative frequency plot of gephyrin cluster position along axons (control, black, *n* = 1585 clusters; KCl, blue, dashed, *n* = 1686 clusters). Distributions not significantly different (*p* > 0.05). **(J)** Gephyrin cluster ratio (in AIS/before AIS) is reduced in KCl-treated neurons, (control: 1.9 ± 0.1; KCl: 0.8 ± 0.1, *p* = 0.008, *n* = 5 preps). Under control conditions, axons contained on average 2.5 ± 0.4 gephyrin clusters before their AIS and 3.8 ± 0.5 clusters within their AIS; in KCl treated neurons, axons contained 3.6 ± 0.6 clusters before their AIS and 3.2 ± 0.5 within their AIS. **(K)** Example neuron stained for ankG and VGAT (labeled as in **A**). Top: control, bottom, KCl. Scale bar = 10 μm. **(L)** Total number of VGAT puncta per axon is not significantly different (*p* > 0.05, *n* = 5). **(M)** Cumulative frequency plot of VGAT puncta position along axon (control, black, *n* = 2219 clusters; KCl, blue, dashed, *n* = 1962 clusters). Distributions not significantly different (*p* > 0.05), suggesting that the tight coupling between pre- and post-inhibitory synapses is not affected by chronic depolarization. **(N)** Cluster ratio (in AIS/before AIS) is lower in KCl treated neurons, (control: 1.9 ± 0.1; KCl: 0.8 ± 0.1, *p* = 0.003, *n* = 5 experiments). Under control conditions, axons contained on average 1.7 ± 0.3 VGAT puncta before their AIS and 3.1 ± 0.5 puncta within their AIS; in KCl treated neurons, axons contained 3.3 ± 0.2 puncta before their AIS and 2.7 ± 0.5 within their AIS.

Recent studies have shown that the AIS can undergo activity-dependent structural plasticity (Kole and Stuart, 2012), and that the AIS can shift distally along the axon in response to chronic depolarization (Grubb and Burrone, 2010). However, whether GABAergic synapses made onto axons (at axo-axonic synapses) also move distally, or adapt to changes in activity, remains unknown. We studied these synapses both under control conditions and after chronic depolarization (15 mM KCl, 48 h, Grubb and Burrone, 2010) by using immunostaining for key GABAergic synapse components (α2, gephyrin, VGAT) and co-staining with ankG (Figures 2A,A'). As previously demonstrated (Grubb and Burrone, 2010), chronic depolarization caused a distal shift in AIS start position (control AIS start position: 8.0 ± 0.8 μm from soma, KCl: 12.3 ± 0.9 μm, *p* = 0.002, Figure 2B) while AIS length was unaffected (*p* > 0.05, Figure 2B'). In contrast to AIS translocation, we found no difference in the number (control: 8.4 ± 0.6; KCl: 8.7 ± 0.6, *p* > 0.05, Figure 2C), or position of α2-GABA_A_R clusters along axons between control and KCl-treated neurons (Figures 2D,E, *p* > 0.05). In agreement with this, a significant decrease in the ratio of axonal α2-GABA_A_R clusters in AIS/before AIS was seen (control: 2.0 ± 0.2; KCl: 1.2 ± 0.1, *p* = 0.004, Figure 2F), further suggesting that α2-GABA_A_R cluster positions along the axon remain fixed compared to homeostatic AIS repositioning.

Gephyrin, a key scaffold protein of GABA_A_Rs at synapses, is also clustered at AIS synapses (Panzanelli et al., 2011). Immunostaining for gephyrin and ankG showed that gephyrin formed numerous clusters along the AIS, and also further along axons (Figure 2G). As for GABA_A_R clusters, we found that the number (Figure 2H) and position (Figure 2I) of gephyrin scaffolds was unaffected by chronic depolarization, leading to a significant decrease in the ratio of gephyrin clusters in AIS/before AIS (Figure 2J). We then used staining for the vesicular GABA transporter VGAT to investigate whether the positioning of presynaptic terminals along the axon was similarly unaffected by chronic depolarization. Similarly, we found no change in the position of GABAergic presynaptic terminals (Figures 2K–N). To confirm that clusters of GABAergic synaptic components found along axons represented bona fide GABAergic synapses, we performed co-labeling for GABA_A_Rs (γ2 subunit) and VGAT (Supplementary Figure 3). We found that a high proportion (85%) of GABA_A_R clusters along the axon were closely opposed to VGAT clusters, and that this value was similar to that for GABA_A_R clusters along dendrites, confirming that GABAergic synapses form along axons. Taken together, these results suggest that the entire GABAergic synapse remains fixed in position during chronic depolarization, and that the tight pre-post coupling of GABAergic synapses (Dobie and Craig, 2011) along the axon is not significantly disrupted during AIS structural plasticity.

### Changes in GABA_A_R cluster size and lateral mobility at the AIS in response to chronic depolarization

**Figure 3:**
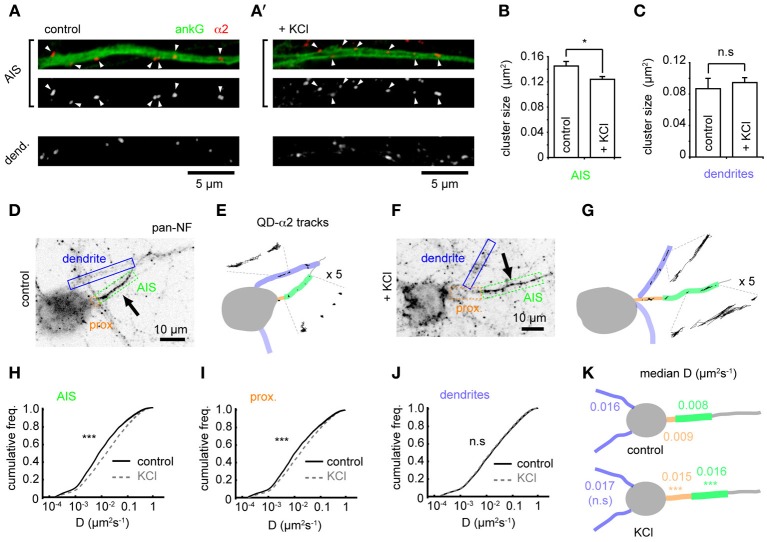
**Changes in GABA_A_R cluster size and lateral mobility at the AIS in response to chronic depolarization. (A)** α2 clustering in AIS and dendrites from control neuron. Top panel: AIS, ankG (green), α2 (red). Arrowheads: clusters in AIS. **(A')** As **(A)**, for KCl-treated neuron. **(B)** α2-GABA_A_R cluster size in the AIS is decreased by KCl treatment (*p* = 0.03, *n* = 5 experiments). (**C)** α2-GABA_A_R cluster size in dendrites is unaffected (*p* > 0.05). **(D)** As Figure 1F. Neurofascin live labeling delineates the AIS (boxed in green). Proximal axon (orange) and dendrites (blue) can also be identified from background staining. **(E)** Map of subcompartments shown in **(A)**, overlaid with QD- α2 tracks. **(F,G)** As in **(C,D)** for KCl-treated neuron. **(H–J)** Instantaneous α2-GABA_A_R diffusion coefficient in control (black) and KCl conditions (gray, dashed) in AIS **(H**), proximal axon **(I)** and dendrites **(J)**. Control, 97 cells; KCl, 111 cells; 10 experiments. In the AIS, median *D* increased 2-fold, *n*_control_ = 40,681, *n*_KCl_ = 49,235, *p* < 2 × 10^−16^; in the proximal axon, median *D* increased 1.7-fold, *n*_control_ = 11,314, *n*_KCl_ = 19,979, *p* < 2 × 10^−16^; in dendrites, lateral mobility was unaffected, *n*_control_ = 316,962, *n*_KCl_ = 463,969, *p* > 0.05. **(K)** Summary of median instantaneous α2-GABA_A_R diffusion coefficient in control and KCl conditions.

Using confocal microscopy we then examined GABA_A_R cluster size in the AIS and dendrites on chronic depolarization. Under control conditions, GABA_A_R clusters were larger in the AIS than dendrites (mean size: AIS, 0.145 ± 0.007 μm^2^; dendrites, 0.087 ± 0.013 μm^2^, *p* = 0.0002, Figures 3A,A'). On chronic depolarization, a small but significant decrease in cluster size was seen in the AIS (control: 0.145 ± 0.007, KCl, 0.124 ± 0.004 μm^2^, *p* = 0.03, Figure 3B). However, dendritic cluster size was slightly but not significantly increased (control, 0.087 ± 0.013, KCl, 0.095 ± 0.006 μm^2^, *p* = 0.2, Figure 3C). Thus, while the position of pre and postsynaptic elements of the GABAergic synapse are uncoupled from activity-dependent AIS translocation, chronic activity drives an AIS-specific reduction in the postsynaptic size of GABAergic synapses.

To investigate if the alteration in α2-GABA_A_R AIS cluster size after chronic activity is due to altered GABA_A_R diffusion dynamics in the AIS, we compared GABA_A_R diffusion dynamics between control and chronically depolarized conditions (Figures 3D–G). Chronic depolarization led to a striking increase in GABA_A_R lateral mobility in the AIS (control-AIS: median *D* = 0.008 μm^2^s^−1^, KCl-AIS: 0.016 μm^2^s^−1^, *p* < 2 × 10^−16^, Figures 3H,K), and GABA_A_R diffusion rates also increased in the proximal axon (between soma and AIS start, control-PA: median *D* = 0.009 μm^2^s^−1^, KCl-PA: 0.015 μm^2^s^−1^, *p* < 2 × 10^−16^, Figures 3I,K). In contrast, GABA_A_R lateral mobility in dendrites was unaffected (control: median *D* = 0.016 μm^2^s^−1^, KCl: 0.017 μm^2^s^−1^, *p* > 0.05, Figures 3J,K). These data suggest that chronic depolarization has a subdomain-specific effect on α2-GABA_A_R diffusion dynamics. Diffusion dynamics of α1-containing GABA_A_Rs in the AIS increased only slightly on chronic depolarization, exhibiting a much smaller change than that seen for α2-GABA_A_Rs in this region (control: median *D* = 0.014 μm^2^s^−1^, KCl: median *D* = 0.017 μm^2^s^−1^, *p* < 2 × 10^−16^, Supplementary Figure 4), and α1-GABA_A_R lateral mobility was unaffected in dendrites (control: median *D* = 0.022 μm^2^s^−1^, KCl: median *D* = 0.022 μm^2^s^−1^, *p* > 0.05). Taken together, these results suggest that the subdomain-specific modulation of GABA_A_R lateral diffusion in response to chronic depolarization primarily affects α2-containing GABA_A_Rs.

### Chronic depolarization affects synaptic and extrasynaptic GABA_A_Rs, and reduces GABA_A_R residency time at AIS synapses

**Figure 4:**
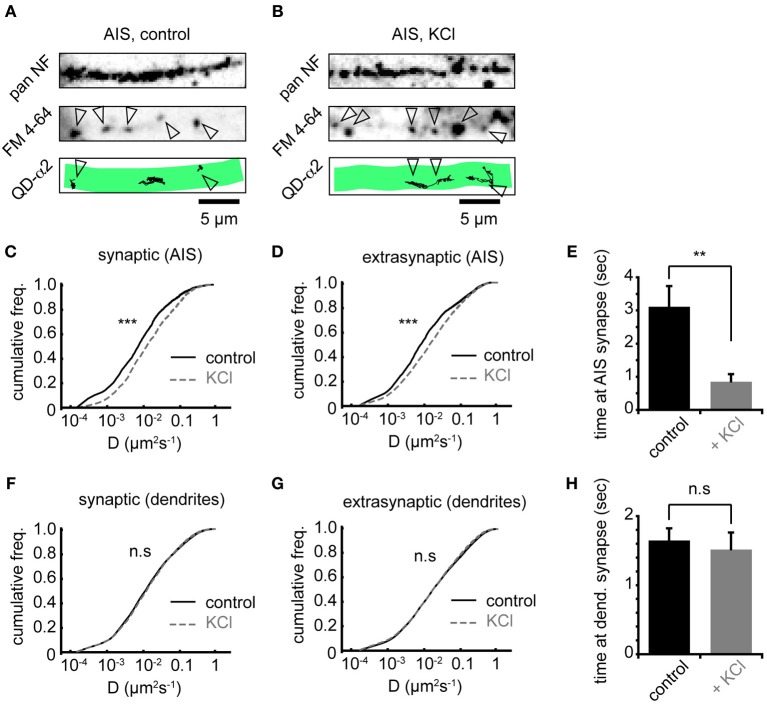
**Chronic depolarization affects synaptic and extrasynaptic GABA_A_Rs, and reduces GABA_A_R residency time at AIS synapses. (A)** Control neuron AIS labeled by pan NF (top), FM 4–64 loading (middle, arrowheads = synapses) and with QD-α2 tracks shown (bottom). **(B)** As in **(A)**, but for KCl-treated neuron. **(C)** Chronic depolarization increases GABA_A_R lateral mobility in the AIS at synapses (1.85-fold increase, *n*_control_ = 1922, *n*_KCl_ = 1620, *p* < 2 × 10^−16^). **(D)** GABA_A_R lateral mobility in the AIS also increases outside synapses (1.89-fold increase, *n*_control_ = 4915, *n*_KCl_ = 8077, *p* < 2 × 10^−16^). **(E)** Mean time spent by GABA_A_Rs at AIS synapses decreases significantly on chronic depolarization (control: *n* = 32 cells; KCl, *n* = 26 cells, *p* = 0.001). **(F,G)** GABA_A_R lateral mobility in dendrites is unaffected (*p* > 0.05) by chronic depolarization, both in synapses, *n*_control_ = 17581, *n*_KCl_ = 13145 **(F)** and outside synapses, *n*_control_ = 75514, *n*_KCl_ = 73135 **(G)**. **(H)** Mean time spent by GABA_A_Rs at synapses in dendrites is unaffected by chronic depolarization (control: *n* = 32 cells; KCl, *n* = 26 cells, *p* > 0.05).

To investigate whether increased AIS-α2-GABA_A_R diffusion dynamics altered receptor behavior at synapses, we labeled presynaptic inputs with FM 4–64. Active presynaptic terminals (FM-positive puncta) were routinely found along neurofascin labeled AISs (Figures 4A,B). Chronic activity increased α2-GABA_A_R diffusion both inside and outside synapses made onto the AIS, with similar increases in each domain (median *D*_syn_ increased 1.8-fold from 0.009 to 0.016 μm^2^s^−1^, Figure 4C; median *D*_ext_ increased 1.8-fold from 0.010 to 0.018 μm^2^s^−1^, Figure 4D, both *p* < 2 × 10^−16^). We also analysed the mean time spent by GABA_A_Rs at synapses. We found that synaptic α2-GABA_A_R residency time at the AIS was significantly decreased (control: 3.1 ± 0.6 s, KCl: 0.9 ± 0.2 s, *p* = 0.001, Figure 4E), suggesting reduced occupancy of synaptic sites, consistent with the decrease in GABA_A_R cluster size observed above. In contrast, chronic depolarization did not affect GABA_A_R lateral mobility in dendrites, either at synapses or outside synapses (median *D*_syn_, control = 0.013 μm^2^s^−1^, KCl = 0.014 μm^2^s^−1^; median *D*_ext_, control = 0.018 μm^2^s^−1^, KCl = 0.018 μm^2^s^−1^; both *p* > 0.05, Mann-Whitney *U*-test, Figures 4F,G). Moreover, mean synaptic residency times for GABA_A_Rs in dendrites were similar between control and KCl conditions (control: 1.6 ± 0.2 s, KCl: 1.5 ± 0.2 s, *p* > 0.05, Figure 4H). Taken together, these data further suggest that chronic activity has a region-specific effect on GABA_A_R diffusion dynamics, with increased diffusion and decreased stability of α2-GABA_A_Rs at AIS synapses upon chronic depolarization.

### Distal shift in AIS position and increased GABA_A_R lateral mobility depend on l-type VGCCs

**Figure 5:**
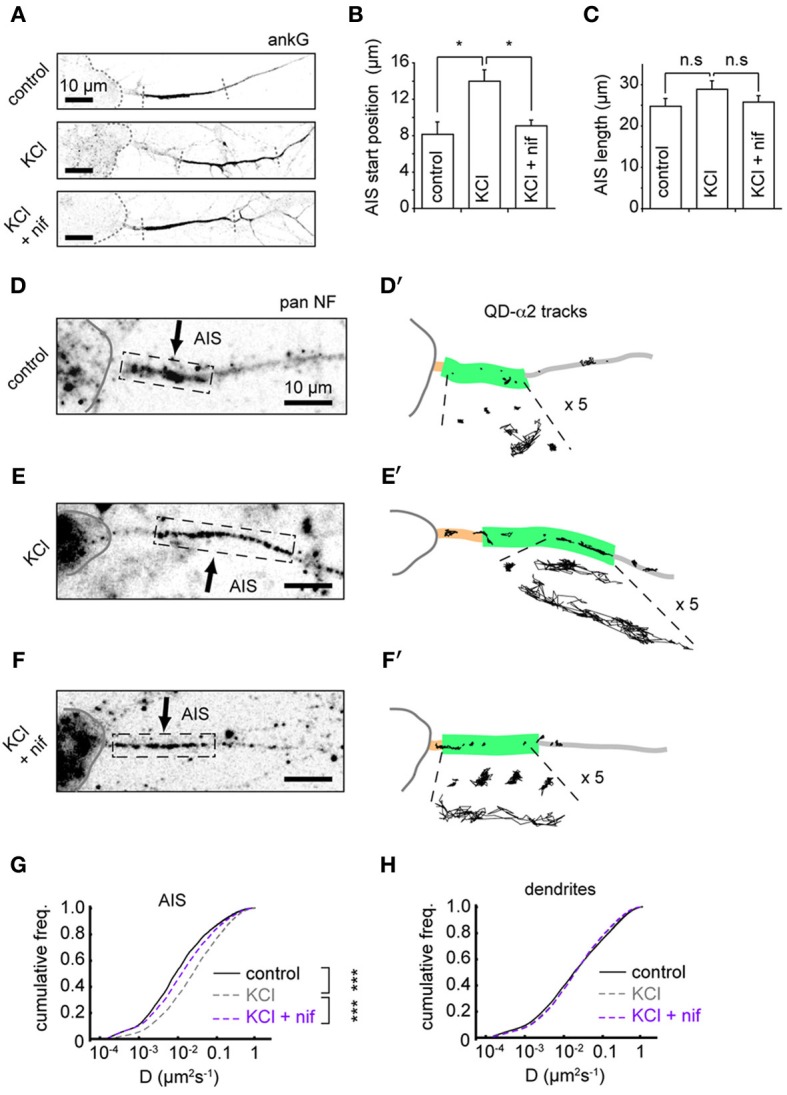
**Distal shift in AIS position and increased GABA_A_R lateral mobility depend on L-type VGCCs. (A)** Example ankG staining from control (top), KCl (middle) and KCl + 5 μM nifedipine conditions (bottom). **(B)** Analysis of AIS start position. One-way ANOVA omnibus test *p* = 0.01. Pairwise test *p*-values are Bonferroni-corrected. KCl treatment caused a distal shift in AIS start position (*p* < 0.05), which was prevented by addition of nifedipine (*p* < 0.05), *n* = 5 experiments (control = 150 cells, KCl = 132 cells and KCl+nif = 116 cells). **(C)** Analysis of AIS length. Omnibus *p* = 0.28. No change in AIS length was seen in either KCl or KCl + nifedipine (*p* > 0.05 for both comparisons). **(D,D')** Example control neuron with AIS location given by pan-NF labeling shown with QD-α2 tracks (those in AIS shown with 5× zoom for clarity). **(E,E')** As above, for KCl treated neuron. **(F,F')** For KCl + nifedipine condition. **(G)** Instantaneous GABA_A_R diffusion coefficient distributions in the AIS for control (black, *n* = 9,482, 17 cells), KCl (gray, dashed, *n* = 15,605, 22 cells) and KCl + nif (purple, dashed, *n* = 6194, 12 cells). Increase in AIS GABA_A_R lateral mobility seen on KCl treatment (*p* < 2 × 10^−16^, Mann-Whitney *U*-test) was reduced in presence of nifedipine, (*p* < 2 × 10^−16^, Mann-Whitney *U*-test). **(H)** As in **(D)**, but for dendrites. Control, *n* = 76,005; KCl, *n* = 102,445; KCl + nif, *n* = 55,821. Dendritic GABA_A_R mobilities are similar across conditions.

To further understand the mechanisms underlying changes in AIS-GABA_A_R diffusion dynamics, we investigated the role of L-type voltage-gated Ca^2+^ channels (VGCCs), whose activity was previously reported to drive activity-dependent AIS translocation (Grubb and Burrone, 2010). We tested whether the L-type calcium channel blocker nifedipine (5 μM) could prevent both the distal AIS shift and the increase in GABA_A_R lateral mobility at the AIS. Immunostaining for ankG confirmed that blockade of L-type VGCCs could indeed prevent AIS translocation (Figures 5A–C). In agreement with the literature, we found that the shift in AIS start position on chronic depolarization was prevented by nifedipine treatment (AIS start position, control: 8.2 ± 1.3 μm; KCl: 14.0 ± 1.2 μm; KCl + nifed: 9.1 ± 0.6 μm, *p* < 0.05 (control vs. KCl), *p* < 0.05 (KCl vs. KCl + nifed), *p* > 0.05 (control vs. KCl + nifed) (Figure 5B). Moreover, no change in AIS length was found under either condition (control: 22.8 ± 1.8 μm; KCl: 28.9 ± 2.0 μm; KCl + nifed: 25.8 ± 1.5 μm, *p* > 0.05 for all comparisons, Figure 5C). We then analysed α2-GABA_A_R diffusion dynamics under these conditions (Figures 5D,D', E,E',F,F'). The robust increase in α2-GABA_A_R diffusion in the AIS upon chronic depolarization (median *D*, control: 0.009 μm^2^s^−1^; KCl: 0.023 μm^2^s^−1^, *p* < 2 × 10^−16^, Mann-Whitney *U*-test) was greatly reduced upon nifedipine treatment (median *D*, KCl + nif: 0.014 μm^2^s^−1^, an 1.64-fold reduction from KCl alone, *p* < 2 × 10^−16^, Mann-Whitney *U*-test, Figure 5G). α2-GABA_A_R lateral mobility in dendrites was similar across control, KCl and KCl + nifedipine conditions (median *D* control, 0.019; KCl, 0.020; KCl + nif, 0.020 μm^2^s^−1^, Figure 5H). Thus, Ca^2+^ influx through L-type VGCCs controls both an activity-dependent shift in AIS location and increased AIS-GABA_A_R lateral mobility.

## Discussion

In this study, we have investigated the surface behavior of GABA_A_Rs at the AIS, both under baseline conditions and in response to changes in neuronal activity that drive AIS structural plasticity (Grubb and Burrone, 2010). We find that surface GABA_A_Rs are less mobile at the AIS than in dendrites, but that chronic depolarization drives increased GABA_A_R lateral mobility and decreased synaptic residency time at the AIS. Intriguingly, both the distal shift in AIS position and increase in GABA_A_R diffusion dynamics at the AIS depend on L-type VGCC activation, suggesting that these activity-dependent responses are linked.

Virtually nothing is known about the behavior of GABA_A_Rs at the AIS. Indeed, to our knowledge, this is the first study to look at the surface trafficking of GABA_A_Rs specifically in the AIS. Our investigation into the clustering and lateral mobility of α1- or α2-containing GABA_A_Rs revealed interesting differences between receptors containing the two subunits. We find that α2-GABA_A_Rs are more numerous in the axon (as shown previously by immunogold electron microscopy, Nusser et al., 1996), and are also found distributed further down the axon, detectable in clusters 100 μm away from the soma. Moreover, α2-GABA_A_Rs are less mobile in the surface membrane than α1-containing GABA_A_Rs, especially at the AIS. Differences in the membrane dynamics of receptors containing these two subunits may be due to GABA_A_R targeting mechanisms that are subunit-specific.

We also find that α2-GABA_A_Rs at the AIS and proximal axon are far less mobile than those in dendrites (which are approximately twice as mobile as their AIS-localized counterparts). Similarly, GABA_A_R cluster size in the AIS is almost twice that in dendrites (Figures 3A–C), and GABA_A_R residency time at synapses in the AIS is longer than for GABA_A_Rs in dendrites (Figures 4E,H). These findings suggest that α2-GABA_A_Rs are especially stable at AIS synapses. Interestingly, GABA_A_Rs also exhibit comparably slower surface dynamics at extrasynaptic sites in the AIS in agreement with the notion that properties of the AIS *per se* may play a role in regulating GABA_A_R mobilities in this neuronal subcompartment. Slow surface dynamics at the AIS have been previously reported for lipids (Nakada et al., 2003) and NaV channels (Brachet et al., 2010). This could in part be due to the high density of protein scaffolds and membrane proteins at the AIS (Rasband, 2010). However, it is additionally possible that specific protein interactions between the α2 subunit and ankG or another AIS protein (e.g., neurofascin 186, which can stabilize axo-axonic synapses, Kriebel et al., 2011) may also act as diffusion traps to contribute to the increased stability of GABA_A_Rs at the AIS (i.e., low diffusion rate, high residency time and cluster size). The gephyrin scaffold can interact directly with the α1, α2, and α3 subunits (Tretter et al., 2008; Mukherjee et al., 2011; Tretter et al., 2011) and forms clusters at the AIS (Panzanelli et al., 2011; also herein, Figure 2G) suggesting that a complex between GABA_A_Rs, gephyrin and AIS proteins may also exist in this region.

The distal shift undergone by the AIS in response to chronic depolarization (Grubb and Burrone, 2010; also observed herein) is an intriguing cell biological phenomenon, for which a molecular mechanism remains unclear. It was recently identified that an ankyrin-B based scaffold in the distal axon can define the position of the AIS (Galiano et al., 2012), which could be involved in AIS structural plasticity. Whether creation and insertion of new axon from the soma is required is also currently unknown. In contrast to the movement of the AIS, we find that GABAergic synapses distributed along the axon do not undergo a distal shift, as the positioning of pre- and postsynaptic components tested (GABA_A_Rs, gephyrin, VGAT) was found to be unaffected by chronic depolarization. While it is unclear how this may affect the ability of these inputs to regulate the initiation of APs, one possibility is that the resulting increase in the number of synaptic inputs between the soma and the shifted AIS could lead to higher inhibitory shunt acting on conductances reaching the AIS. This would raise the threshold for AP initiation, counterbalancing the chronic activity stimulus and thus acting homeostatically, in concert with the distal shift in AIS position, which causes increased thresholds for action potential initiation (Grubb and Burrone, 2010; O'Leary et al., 2010). Activity-dependent disruption of scaffolding interactions may underlie the observed increase in GABA_A_R diffusion and decrease in GABA_A_R cluster size at the AIS. Since the activity-dependent increase in GABA_A_R mobility at the AIS is also seen extrasynaptically (in gephyrin negative regions) we think it unlikely that alterations in gephyrin-dependent GABA_A_R stabilization are the primary driver of the increase in GABA_A_R mobility at the AIS upon chronic depolarization. Rather, a distal shift in AIS position but not GABAergic synapses may uncouple GABA_A_Rs from mechanisms that contribute to their stabilization in the axonal membrane. An intriguing possibility is that the AIS-specific mechanisms that stabilize GABA_A_Rs in the axon may be weakened in order to allow GABAergic synapses to remain fixed in position and resist the distal shift of the AIS scaffold (including neurofascin 186) in response to chronic depolarization. Increased GABA_A_R diffusion dynamics in the AIS and proximal axon could be a necessary consequence of such reduced tethering, to allow the preservation of GABAergic synaptic positions along the axon. Moreover, these putative interactions could be disrupted by activation of L-type VGCCs, since inhibition of L-type VGCCs with nifedipine blocks translocation of the AIS, and also partially prevents an increase in AIS-GABA_A_R diffusion on KCl treatment. Previous studies revealed that acute increases in neuronal activity and spiking (e.g., driven by treatment with 4-AP or glutamate) lead to rapid calcium and calcineurin-dependent GABA_A_R de-clustering and increased GABA_A_R diffusion dynamics in dendrites (Bannai et al., 2009; Muir et al., 2010). In contrast we found that chronic treatment with low levels of KCl (15 mM, 48 h), which was shown to cause only a small 10mV depolarization of the resting membrane potential and a suppression of spontaneous spiking (Grubb and Burrone, 2010) only increased GABA_A_R diffusion at the AIS but not in dendrites, suggesting that mild chronic depolarization (with KCl) cannot drive a sufficient rise in dendritic calcium to activate dendritic calcineurin or alter dendritic GABA_A_R stability. Interestingly, chronic KCL-dependent AIS repositioning was also recently demonstrated to be calcineurin-dependent suggesting that these conditions may lead to a selective increase in somatic and/or AIS specific calcineurin activity (Evans et al., 2013). This could also account (perhaps in concert with the localization of a specific scaffold such as AnkG to the proximal axon) for a more localized activity-dependent impact on GABA_A_R diffusion in the proximal axon/AIS (rather than throughout the entire axon). It will be interesting to determine in the future if the activity-dependent increase in GABA_A_R mobility at the AIS is also dependent on changes in GABA_A_R phosphorylation state (Muir et al., 2010).

While GABAergic inputs onto the AIS are ideally localized to control action potential initiation (Kole and Stuart, 2012), the nature of these inputs, i.e., whether they are inhibitory or excitatory, is still unresolved. A body of evidence suggests that GABAergic inputs onto the AIS can be depolarizing in the cortex (Szabadics et al., 2006; Khirug et al., 2008; Kole and Stuart, 2012). This is thought to be due to high expression of the Na^+^/K^+^/Cl^−^ cotransporter NKCC1 (Khirug et al., 2008) and absence of the K^+^/Cl^−^ cotransporter KCC2 from the AIS (Hedstrom et al., 2008; Báldi et al., 2010), resulting in a high intracellular [Cl^−^] and subsequent depolarization on GABA_A_R activation. Thus, reduced GABA_A_R cluster size and increased GABA_A_R diffusion at AIS synapses in response to chronic depolarization could alternatively represent weakening of depolarizing or excitatory GABAergic inputs. In this case, increased GABA_A_R diffusion dynamics would provide a mechanism to weaken depolarizing inputs in a homeostatic response to chronic elevation of activity.

We conclude that during activity-dependent AIS translocation, occurring in response to chronic depolarization, the positions of GABAergic synapses along the axon are unaffected. However, the AIS shift is coupled with plasticity of GABA_A_R cluster size and diffusion dynamics at this key neuronal subcompartment. This novel form of plasticity could be important for GABAergic control of information processing in the healthy or diseased brain, for example in epilepsy, where repeated bursts of activity may lead to structural plasticity of the AIS and of axonal GABA_A_R diffusion dynamics.

### Conflict of interest statement

The authors declare that the research was conducted in the absence of any commercial or financial relationships that could be construed as a potential conflict of interest.

## Supplementary material

Supplementary Figure 1 **(A)** Schematic showing live-labeling of AIS via an antibody to neurofascin. We used an antibody to an extracellular epitope on neurofascin (NF), pre-conjugated to alexa dye. **(B)** Overlap of pan-NF live labeling with AIS as marked by ankG-GFP, confirming that this approach can reliably label the AIS. Scale bar = 10 μm.

Supplementary Figure 2 **(A)** mGFP-transfected neuron. Left: ankG staining (AIS); right: mGFP expression, with AIS and sample dendrite labeled. **(B)** Zoomed regions of AIS and dendrite shown boxed in **(A)**. **(C)** Process diameter of AIS and dendrites is not significantly different. AIS: 1.0 ± 0.1 μm (*n* = 14), dendrite: 1.1 ± 0.1 μm (*n* = 28), *p* = 0.54).

Supplementary Figure 3 **(A)** Axon from neuron stained for ankG (top, cyan), γ2-GABA_A_Rs (middle, magenta) and VGAT (bottom, yellow), shown merged beneath. Closed arrowheads indicate position of a GABA_A_R cluster opposed to a VGAT cluster; open arrowheads indicate position of a GABA_A_R cluster not opposed to a VGAT cluster. (**B)** Synaptic GABA_A_R cluster fraction in axons and dendrites is not significantly different. Axon: 0.86 ± 0.03 (*n* = 30), dendrite: 0.80 ± 0.01 (*n* = 30), *p* = 0.08.

Supplementary Figure 4 **(A)** As Figure 1C. Subcompartments delineated from neurofascin staining overlaid with α1-QD trajectories. **(B)** As **(A)**, for KCl-treated neuron. **(C,D)** Instantaneous α1-GABA_A_R diffusion coefficient in control (black) and KCl conditions (gray, dashed) in AIS **(C)** and dendrites **(D)**. Control, 45 cells; KCl, 47 cells; 5 experiments. In the AIS, median *D* increased 1.2-fold, *n*_control_ = 10,099, *n*_KCl_ = 18,933, *p* < 2 × 10^−16^; in dendrites, lateral mobility was unaffected, *n*_control_ = 137,377, *n*_KCl_ = 175,592, *p* > 0.05.

## References

[B1] Báldi R. Varga C. Támas G. (2010). Differential distribution of KCC2 along the axo-somato-dendritic axis of hippocampal principal cells. Eur. J. Neurosci. 32, 1319–1325 10.1111/j.1460-9568.2010.07361.x 20880357

[B2] Banker G. Goslin G. (1991). Culturing Nerve Cells, 2nd Edn. Cambridge, MA: MIT press

[B3] Bannai H. Lévi S. Schweizer C. Inoue T. Launey T. Racine V. (2009). Activity-dependent tuning of inhibitory neurotransmission based on GABA_A_R diffusion dynamics. Neuron 62, 670–682 10.1016/j.neuron.2009.04.023 19524526

[B5] Brachet A. Leterrier C. Irondelle M. Fache M.-P. Racine V. Sibarita J.-B. (2010). Ankyrin G restricts ion channel diffusion at the axonal initial segment before the establishment of the diffusion barrier. J. Cell Biol. 191, 383–395 10.1083/jcb.201003042 20956383 PMC2958486

[B6] Brünig I. Scotti E. Sidler C. Fristchy J.-M. (2001). Intact sorting, targeting, and clustering of γ-aminobutyric acid A receptor subtypes in hippocampal neurons *in vitro*. J. Comp. Neurol. 443, 43–55 10.1002/cne.10102 11793346

[B7] Dobie F. A. Craig A. M. (2011). Inhibitory synapse dynamics: coordinated presynaptic and postsynaptic mobility and the major contribution of recycled vesicles to new synapse formation. J. Neurosci. 31, 10481–10493 10.1523/JNEUROSCI.6023-10.2011 21775594 PMC6622636

[B8] Evans M. D. Sammons R. P. Lebron S. Dumitrescu A. S. Watkins T. B. Uebele V. N. (2013). Calcineurin signaling mediates activity-dependent relocation of the axon initial segment. J. Neurosci. 33, 6950–6963 10.1523/JNEUROSCI.0277-13.2013 23595753 PMC3743026

[B9] Galiano M. R. Jha S. Ho T. S. Y. Zhang C. Ogawa Y. Chang K.-J. (2012). A distal axonal cytoskeleton forms an intra-axonal boundary that controls axon initial segment assembly. Cell 149, 1125–1139 10.1016/j.cell.2012.03.039 22632975 PMC3361702

[B10] Grubb M. S. Burrone J. (2010). Activity-dependent relocation of the axon initial segment fine-tunes neuronal excitability. Nature 465, 1070–1074 10.1038/nature09160 20543823 PMC3196626

[B11] Hedstrom K. L. Ogawa Y. Rasband M. N. (2008). AnkyrinG is required for maintenance of the axon initial segment and neuronal polarity. J. Cell Biol. 183, 635–640 10.1083/jcb.200806112 19001126 PMC2582894

[B12] Khirug S. Yamada J. Afzalov R. Voipio J. Khiroug L. Kaila K. (2008). GABAergic depolarization of the axon initial segment in cortical principal neurons is caused by the Na-K-2Cl cotransporter NKCC1. J. Neurosci. 28, 4635–4639 10.1523/JNEUROSCI.0908-08.2008 18448640 PMC6670448

[B12a] Klausberger T. Somogyi P. (2008). Neuronal diversity and temporal dynamics: the unity of hippocampal circuit operations. Science 321, 53–57 10.1126/science.1149381 18599766 PMC4487503

[B13] Kole M. H. P. Stuart G. J. (2012). Signal processing in the axon initial segment. Neuron 73, 235–247 10.1016/j.neuron.2012.01.007 22284179

[B14] Kriebel M. Metzger J. Trinks S. Chugh D. Harvey R. J. Harvey K. (2011). The cell adhesion molecule neurofascin stabilizes axo-axonic GABAergic terminals at the axon initial segment. J. Biol. Chem. 286, 24385–24393 10.1074/jbc.M110.212191 21576239 PMC3129217

[B16] Luscher B. Fuchs T. Kilpatrick C. L. (2011). GABA_A_ receptor trafficking-mediated plasticity of inhibitory synapses. Neuron 70, 385–409 10.1016/j.neuron.2011.03.024 21555068 PMC3093971

[B17] Muir J. Arancibia-Carcamo I. L. Macaskill A. F. Smith K. R. Griffin L. D. Kittler J. T. (2010). NMDA receptors regulate GABA_A_ receptor lateral mobility and clustering at inhibitory synapses through serine 327 on the γ2 subunit. Proc. Natl. Acad. Sci. U.S.A. 107, 16679–16684 10.1073/pnas.1000589107 20823221 PMC2944765

[B18] Mukherjee J. Kretschmannova K. Gouzer G. Maric H.-M. Ramsden S. Tretter V. (2011). The residence time of GABA_A_Rs at inhibitory synapses is determined by direct binding of the receptor α1 subunit to gephyrin. J. Neurosci. 31, 14677–14687 10.1523/JNEUROSCI.2001-11.2011 21994384 PMC3202462

[B19] Nakada C. Ritchie K. Oba Y. Nakamura M. Hotta Y. Iino R. (2003). Accumulation of anchored proteins forms membrane diffusion barriers during neuronal polarization. Nat. Cell Biol. 5, 626–632 10.1038/ncb1009 12819789

[B20] Nusser Z. Sieghart W. Benke D. Fritschy J. M. Somogyi P. (1996). Differential synaptic localization of two major γ-aminobutyric acid type A receptor α subunits on hippocampal pyramidal cells. Proc. Natl. Acad. Sci. U.S.A. 93, 11939–11944 10.1073/pnas.93.21.11939 8876241 PMC38162

[B21] O'Leary T. van Rossum M. C. W. Wyllie D. J. A. (2010). Homeostasis of intrinsic excitability in hippocampal neurones: dynamics and mechanism of the response to chronic depolarization. J. Physiol. (Lond.) 588, 157–170 10.1113/jphysiol.2009.181024 19917565 PMC2821556

[B22] Panzanelli P. Gunn B. G. Schlatter M. C. Benke D. Tyagarajan S. K. Scheiffele P. (2011). Distinct mechanisms regulate GABA_A_ receptor and gephyrin clustering at perisomatic and axo-axonic synapses on CA1 pyramidal cells. J. Physiol. (Lond.) 589, 4959–4980 10.1113/jphysiol.2011.216028 21825022 PMC3224886

[B23] Rasband M. N. (2010). The axon initial segment and the maintenance of neuronal polarity. Nat. Rev. Neurosci. 11, 552–562 10.1038/nrn2852 20631711

[B24] Renner M. Domanov Y. Sandrin F. Izeddin I. Bassereau P. Triller A. (2011). Lateral diffusion on tubular membranes: quantification of measurements bias. PLoS ONE 6:e25731 10.1371/journal.pone.0025731 21980531 PMC3183067

[B25] Schafer D. P. Jha S. Liu F. Akella T. McCullough L. D. Rasband M. N. (2009). Disruption of the axon initial segment cytoskeleton is a new mechanism for neuronal injury. J. Neurosci. 29, 13242–13254 10.1523/JNEUROSCI.3376-09.2009 19846712 PMC2801423

[B26] Szabadics J. Varga C. Molnár G. Oláh S. Barzó P. Tamás G. 2006 Excitatory effect of GABAergic axo-axonic cells in cortical microcircuits. Science 311, 233–235 10.1126/science.1121325 16410524

[B27] Thomas P. Mortensen M. Hosie A. M. Smart T. G. (2005). Dynamic mobility of functional GABA_A_ receptors at inhibitory synapses. Nat. Neurosci. 8, 889–897 10.1038/nn1483 15951809

[B28] Tretter V. (2011). Molecular basis of the GABA_A_ receptor α3 subunit interaction with gephyrin. J. Biol. Chem. 31, 37702–37711 10.1074/jbc.M111.291336 21880742 PMC3199513

[B29] Tretter V. Jacob T. C. Mukherjee J. Fritschy J.-M. Pangalos M. N. Moss S. J. (2008). The clustering of GABA_A_ receptor subtypes at inhibitory synapses is facilitated via the direct binding of receptor α2 subunits to gephyrin. J. Neurosci. 28, 1356–1365 10.1523/JNEUROSCI.5050-07.2008 18256255 PMC6671568

[B30] Twelvetrees A. E. Yuen E. Y. Arancibia-Carcamo I. L. MacAskill A. F. Rostaing P. Lumb M. J. (2010). Delivery of GABA_A_Rs to synapses is mediated by HAP1-KIF5 and disrupted by mutant huntingtin. Neuron 65, 53–65 10.1016/j.neuron.2009.12.007 20152113 PMC2841506

