# Supplementary material for: Plasticity of GABAA receptor diffusion dynamics at the axon initial segment
Source: Front Cell Neurosci. 2014 Jun 10;8:151. doi: 10.3389/fncel.2014.00151 (PMC4051194; doi:10.3389/fncel.2014.00151)
Supplement: Supplementary Figure 1 — (A) Schematic showing live-labeling of AIS via an antibody to neurofascin. We used an antibody to an extracellular epitope on neurofascin (NF), pre-conjugated to alexa dye. (B) Overlap of pan-NF live labeling with AIS as marked by ankG-GFP, confirming that this approach can reliably label the AIS. Scale bar = 10 μm. [file DataSheet1.ZIP › Datasheet1.pdf]

Receptor subunit	Location	Median D / um2s-1		Reference
		Control	KCl	
α2	AIS	0.008	0.016	Fig 1, 3
	Proximal axon	0.009	0.015	
	Dendrites	0.016	0.017	
α1	AIS	0.014	0.017	Fig 1, 3
	Dendrites	0.022	0.022	
α2	AIS, synaptic (+FM)	0.009	0.016	Fig 4
	AIS, extrasynaptic (-FM)	0.010	0.018	
	Dendrite, synaptic (+FM)	0.013	0.014	
	Dendrites,extrasynaptic (-FM)	0.018	0.018	
α2	AIS, control	0.009		Fig 5
	AIS, KCl	0.023		
	AIS, KCl + nifedipine	0.014		
