# Supplementary figures and images for: Plasticity of GABAA receptor diffusion dynamics at the axon initial segment
Source: Front Cell Neurosci. 2014 Jun 10;8:151. doi: 10.3389/fncel.2014.00151 (PMC4051194; doi:10.3389/fncel.2014.00151)

# Supplementary Figure 1 - Muir et al.

**A**

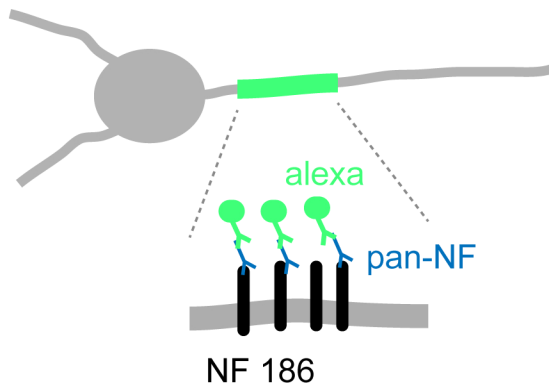

**B**

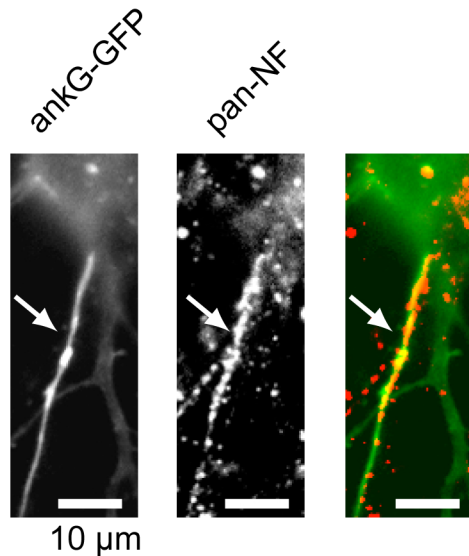

Supplement: Supplementary Figure 1 — (A) Schematic showing live-labeling of AIS via an antibody to neurofascin. We used an antibody to an extracellular epitope on neurofascin (NF), pre-conjugated to alexa dye. (B) Overlap of pan-NF live labeling with AIS as marked by ankG-GFP, confirming that this approach can reliably label the AIS. Scale bar = 10 μm. [file DataSheet1.ZIP › S1.pdf]

# Supplementary Figure 2 - Muir et al.

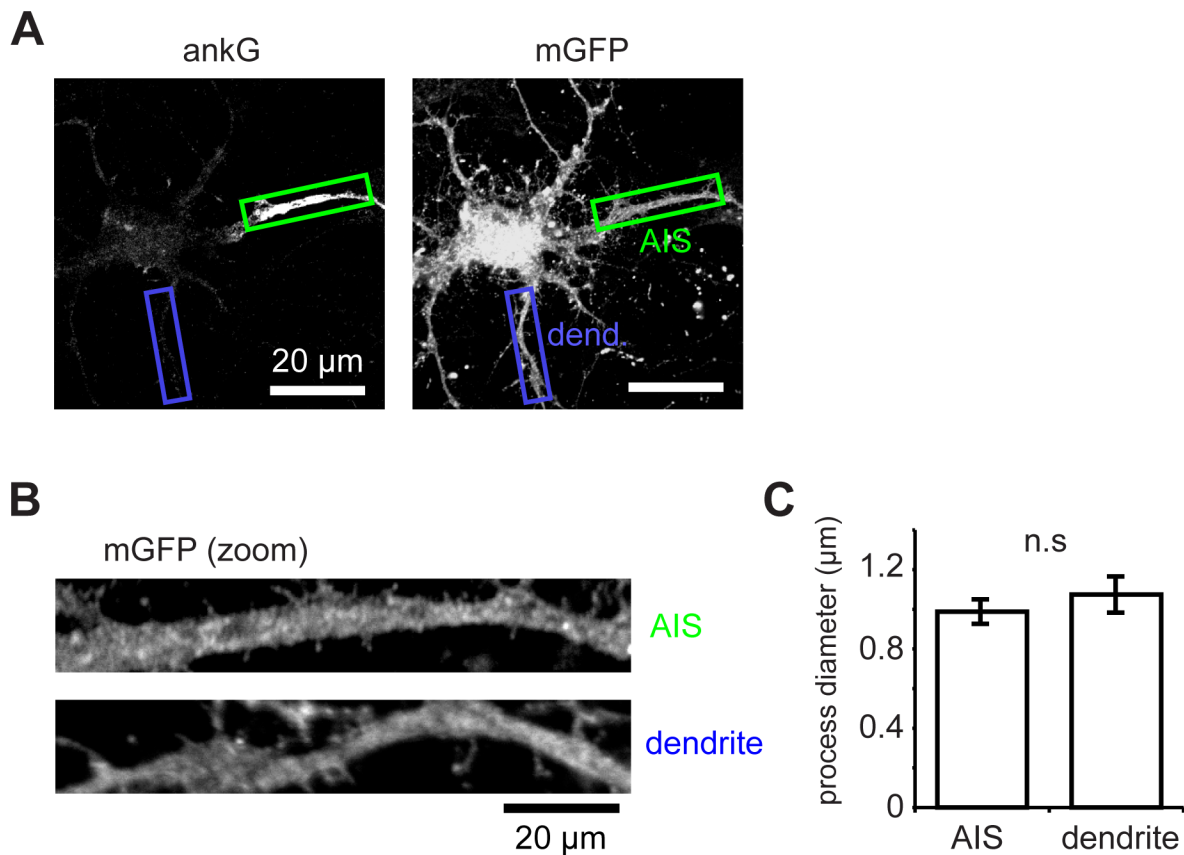

Supplement: Supplementary Figure 1 — (A) Schematic showing live-labeling of AIS via an antibody to neurofascin. We used an antibody to an extracellular epitope on neurofascin (NF), pre-conjugated to alexa dye. (B) Overlap of pan-NF live labeling with AIS as marked by ankG-GFP, confirming that this approach can reliably label the AIS. Scale bar = 10 μm. [file DataSheet1.ZIP › S2.pdf]

# Supplementary Figure 3 - Muir et al.

**A**

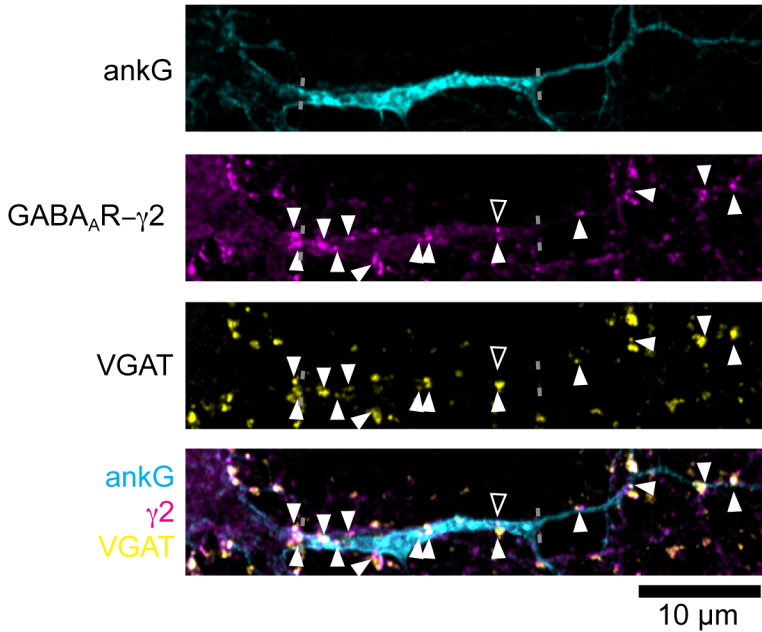

**B**

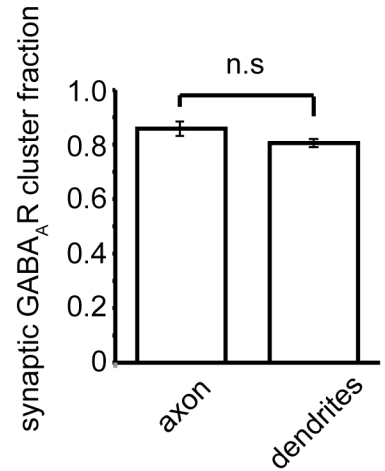

Supplement: Supplementary Figure 1 — (A) Schematic showing live-labeling of AIS via an antibody to neurofascin. We used an antibody to an extracellular epitope on neurofascin (NF), pre-conjugated to alexa dye. (B) Overlap of pan-NF live labeling with AIS as marked by ankG-GFP, confirming that this approach can reliably label the AIS. Scale bar = 10 μm. [file DataSheet1.ZIP › S3.pdf]

# Supplementary Figure 4 - Muir et al.

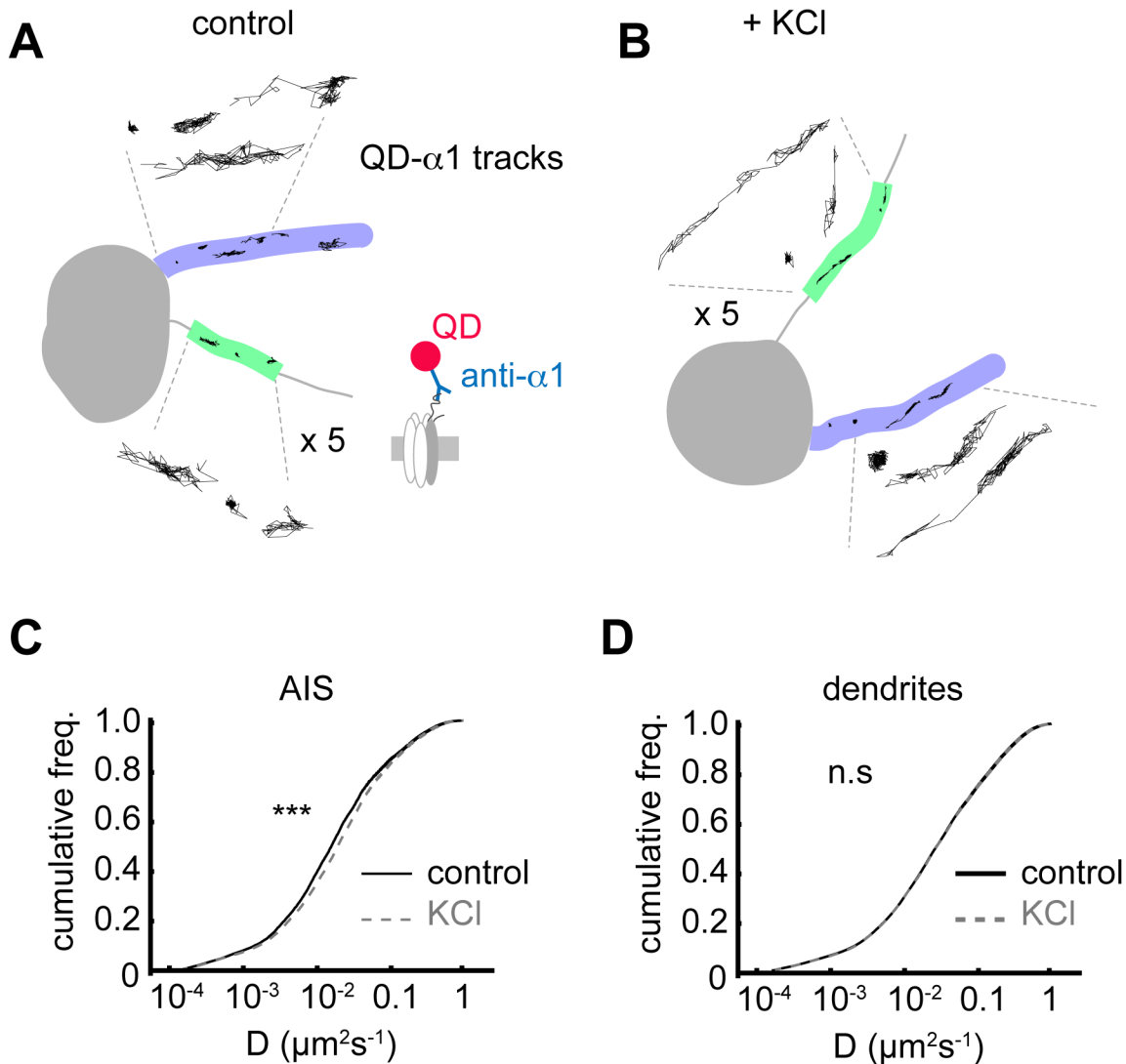

Supplement: Supplementary Figure 1 — (A) Schematic showing live-labeling of AIS via an antibody to neurofascin. We used an antibody to an extracellular epitope on neurofascin (NF), pre-conjugated to alexa dye. (B) Overlap of pan-NF live labeling with AIS as marked by ankG-GFP, confirming that this approach can reliably label the AIS. Scale bar = 10 μm. [file DataSheet1.ZIP › S4.pdf]
